# Induced Expression of Nucleolin Phosphorylation-Deficient Mutant Confers Dominant-Negative Effect on Cell Proliferation

**DOI:** 10.1371/journal.pone.0109858

**Published:** 2014-10-14

**Authors:** Shu Xiao, Elif Caglar, Priscilla Maldonado, Dibash Das, Zaineb Nadeem, Angela Chi, Benjamin Trinité, Xin Li, Anjana Saxena

**Affiliations:** 1 Biology Department, Brooklyn College, Brooklyn, New York, United States of America; 2 City University of New York, Graduate Center, New York, New York, United States of America; 3 New York University School of Medicine, New York, New York, United States of America; 4 Great Neck South High School, Great Neck, New York, United States of America; 5 New York University College of Dentistry, New York, New York, United States of America; Virginia Commonwealth University, United States of America

## Abstract

Nucleolin (NCL) is a major nucleolar phosphoprotein that has pleiotropic effects on cell proliferation and is elevated in a variety of tumors. NCL is highly phosphorylated at the N-terminus by two major kinases: interphase casein kinase 2 (CK2) and mitotic cyclin-dependent kinase 1 (CDK1). Earlier we demonstrated that a NCL-mutant that is partly defective in undergoing phosphorylation by CK2 inhibits chromosomal replication through its interactions with Replication Protein A, mimicking the cellular response to DNA damage. We further delineated that the N-terminus of NCL associates with Hdm2, the most common E3 ubiquitin ligase of p53. We reported that NCL antagonizes Hdm2 to stabilize p53 and stimulates p53 transcriptional activity. Although NCL-phosphorylation by CK2 and ribosomal DNA transcription are closely coordinated during interphase, the role of NCL phosphorylation in regulating cell proliferation remains unexplored. We have therefore engineered unique human cells that specifically induce expression of NCL-wild type (WT) or a phosphorylation-deficient NCL-mutant, 6/S*A where all the six CK2 consensus serine sites residing in the N-terminus NCL were mutated to alanine. Here we show that this NCL-mutant is defective in undergoing phosphorylation by CK2. We also demonstrate that NCL-phosphorylation by CK2 is required through the S-phase progression in cell cycle and hence proliferation. Induced expression of NCL with mutated CK2 phosphorylation sites stabilizes p53, results in higher expression of Bcl2 (B-cell lymphoma 2) homology 3 (BH3)-only apoptotic markers and causes a dominant-negative effect on cell viability. Our unique cellular system thus provides the first evidential support to delineate phospho-specific functions of NCL on cell proliferation.

## Introduction

Nucleolin (NCL or C23), a ubiquitously expressed phosphoprotein constitutes almost 10% of total nucleolar proteins. NCL protein has multiple sub-cellular locations that are directly implicated in its pleotropic physiological functions. In the nucleolus, it is directly involved in cellular processes e.g. chromatin remodeling [1], transcriptional regulation [2], ribosome biogenesis [3] and telomerase activity [4]. In the nucleoplasm, it interacts with several proteins and is involved in regulation of the cellular response to stress [5]–[10]. NCL constantly shuttles between the nucleus and cytoplasm where it is involved in many non nucleolar functions e.g. centrosome duplication [11] as well as post-transcriptional and translational regulation of various mRNAs [12]–[14] including p53 [15]. On the cell surface NCL serves as a receptor, binds to several ligands to either mediate tumorigenesis or to relay anti-carcinogenic effects [16].

Differential NCL localization is due to changes in its isoelectric point and/or post-translational modifications that include: glycosylation [17], ADP-ribosylation [18], acetylation [19] and most importantly phosphorylation [20]–[23]. NCL is a substrate for a variety of kinases and NCL phosphorylation has been implicated in its diverse physiological functions. In exponentially dividing cells, casein kinase 2 (CK2) phosphorylates NCL at the consensus serine sites (typically ‘SEDE’ motifs) residing in its N-terminus [23]–[25]. Increases in CK2 activity and heightened phosphorylation of NCL by CK2 are positively correlated with active rDNA transcription, rRNA synthesis, cell growth and proliferation [20], [26], [27]. During mitosis, cyclin-dependent kinase 1 (CDK1) phosphorylates threonine within ‘TPKK’ motifs that are also located in the N-terminal domain of NCL [21], [28]. In sum, NCL phosphorylation regulates cell cycle and sub-cellular localization that is linked to nucleolar reorganization during mitosis [29], [30]. Besides the phosphorylation sites, the N-terminal domain of NCL also harbors acidic stretches (that interact with histones) and basic regions (that interact with DNA), leading to its role in chromatin decondensation [31]. It can be envisioned that sequential CK2 and CDK1 phosphorylation could modulate NCL function in controlling cell proliferation (growth and division) between interphase and the mitotic phase of the cell cycle.

RNA binding properties of NCL are conferred by its four RNA binding domains that are adjacent to the N-terminus. NCL has been described as a stress responsive RNA binding protein [6]. NCL translocation from nucleoli to nucleoplasm in response to different stresses [5], [32] has been linked to its role in inhibiting DNA replication [7], [33], [34], controlling DNA repair [35], [36] and gene transcription [37]–[39]. Together, these data indicate that NCL mobilization releases a part of the NCL pool to modulate DNA and RNA metabolism following stress.

NCL also has distinct mechanisms in p53 regulation. We previously demonstrated that in cells with hyperproliferative signals but no obvious DNA damage, NCL stabilizes p53 protein [8]. We demonstrated that the N-terminus and central RNA binding domains of NCL associate strongly with Hdm2 to inhibit p53 degradation *in vitro* [9]. However, upon DNA damage a part of the NCL pool can bind p53 mRNA and repress p53 translation [15] while stabilizing Bcl2 (B-cell lymphoma 2) mRNA, a downstream target of the p53 signaling in the cytoplasm [13]. On the other hand, mobilization of NCL upon DNA damage alters NCL interactions with the ribosomal subunit RPL26 (60S ribosomal protein L26) and the p53 antagonist Hdm2 leading to an increase in p53 mRNA translation and protein stability [5], [9], [40]. Thus, NCL protein regulates the p53 signaling pathway at multiple levels, providing a fine-tuning on cell survival during cellular response to stress. Nonetheless, the role of NCL phosphorylation by CK2 on cell survival and proliferation functions remains largely unknown.

We have engineered unique system using human NARF6 cells [41]. The NARF6 cells which were originally derived from human osteosarcoma U2OS (ATCC) cells, express wt-p53 as well as support the IPTG (isopropyl β-D-1-thiogalactopyranoside) -regulated expression of the p14ARF (*A*lternate *R*eading *F*rame), an upstream regulator of p53 during oncogenic stimulation [41]. We have genetically modified NARF6 cells through retroviral infection such that to further support induced expression of either NCL wild type (WT) or a CK2 phosphorylation-deficient mutant (6/S*A; containing six alanine substitutions at the consensus serine sites) by a Tet-off promoter system. Hereafter we refer to these modified cells as NARF6 NCL clones or inducible NCL (WT or 6/S*A) cells. In this study we demonstrate the importance of these six consensus CK2 sites on NCL and demonstrate that CK2 phosphorylation-deficient NCL mutant triggers p53 checkpoint activation and inhibits cell proliferation by activating pro-apoptotic markers.

## Results

### Nucleolin mutations at the CK2 phosphorylation sites significantly reduces its phosphorylation

**Figure 1:**
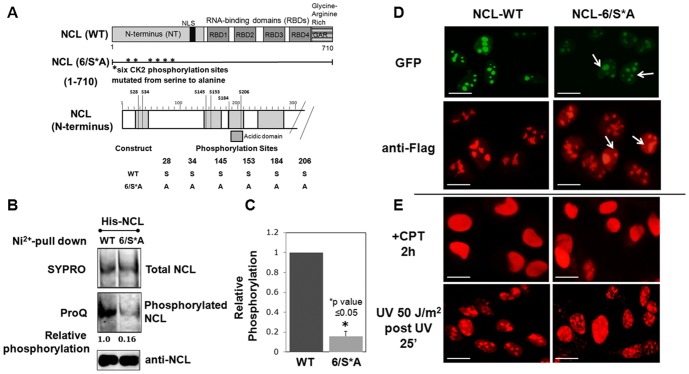
Characterization of phosphorylation-deficient NCL-mutant. **(A) *Targeting the consensus CK2 sites in NCL***: The modular structure of NCL protein is shown, indicating the positions of principal domains. The six consensus CK2 sites (all serine) that were mutated to alanine in the 6/S*A construct are denoted by asterisks (*). An enlarged schematic of the N-terminal domain is also shown. **(B)** Nucleolin (NCL)-6/S*A is hypophosphorylated: Purified His-tagged NCL proteins were subjected to SDS-PAGE and fluorescently stained and visualized for phospho-specific Pro-Q Diamond and SYPRO-Ruby protein (for total NCL) dyes. Anti-NCL is a representative Western blot. NCL-6/S*A relative phosphorylation is represented as the ratio of its Pro-Q signal to that of NCL-WT, after normalizing for the total NCL signal using NIH Image J software. NCL-6/S*A was only phosphorylated at 16% the level of WT, due to its mutated CK2 sites. **(C)** Values in the bar graph are the mean ±S.E.M. from three experiments from ProQ and SYPRO-Ruby staining of NCL-variants. *Statistically different from NCL-WT phosphorylation, p<0.05. **(D)** Sub-nuclear localization of NCL-WT and -6/S*A: Sub-nuclear localization of transiently transfected GFP-NCL (green) and stable inducible 3xFlag-NCL (red) in U2OS cells as detected by anti-Flag antibodies. Under normal (unstressed) conditions, both WT and the 6/S*A mutant are primarily localized to the nucleoli (punctate staining). A significantly larger fraction of nuclear 6/S*A (60.0±4.0%, **p<0.005) was localized in the nucleoplasm as compared to that of WT (which is only at 35.5±8.5% of the total). Nucleoplasmic staining (diffused within nucleus) is shown with white arrows. For quantitation refer to Figure S1. **(E)** ***Sub-nuclear localization of 3xFlag-NCL (red) upon DNA damaging conditions***: NCL (WT and 6/S*A) translocate completely to nucleoplasm upon treatment with topoisomerase I inhibition by camptothecin (CPT, 2 µM for 2 h) while post-UV (50 J m^−2^) at 25 min, both WT and mutant are significantly located in nucleoli as well as nucleoplasm. Scale bar represents 10 µm.

In order to reveal phosphorylation specific NCL functions, we created a NCL-6/S*A construct where the six consensus CK2 sites were mutated from serine to alanine (Figure 1A). To examine the effect of the NCL-6/S*A mutation on phosphorylation, His-tagged NCL-WT or the 6/S*A mutant, expressed in U2OS cells and were purified by affinity chromatography. After SDS-PAGE, proteins were stained with Pro-Q Diamond for quantifying phosphorylated Ser, Thr and Tyr residues [42]. Subsequent staining of the same gel with SYPRO Ruby for total protein content [43] revealed that the NCL-6/S*A mutant was only 16% phosphorylated as compared to WT, demonstrating that the mutation of the six CK2 sites greatly reduces NCL phosphorylation (Figure 1B, 1C, *p<0.05). Anti-NCL is shown in a parallel gel for the Western blot detection. Preliminary studies by our laboratory indicate that the nucleolin phosphorylation by CDK1 however, remains unchanged between NCL-WT and 6/S*A (K. Ng and A. Saxena, unpublished data). These data thus strongly suggest that CK2 is the major kinase that phosphorylates NCL during interphase, confirming an earlier report [21].

### Nucleolin phospho-variants demonstrate distinct sub-nuclear localization and mobility upon genotoxic stress

Earlier we demonstrated that partial dephosphorylation at CK2 sites leads significant fraction of NCL to localize in the nucleoplasm [7], [33], . We therefore examined sub-nuclear localization of NCL-6/S*A. Both WT and the 6/S*A mutant primarily localized to the nucleoli (punctate staining, Figure 1D) upon transient transfection or stable inducible expression (refer to later sections for details regarding inducible cells). Additionally, NCL-6/S*A is also readily localized in the nucleoplasm as compared to WT (Figure 1D). To quantitate the sub-nuclear distribution of NCL localization, we examined a total of ∼80-100 nuclei with differential levels of NCL expression (low, medium and high) in both WT and 6/S*A expressing cells. Integrated morphometric analyses performed in cells with moderate level of NCL expression (n = 30 for each WT and 6/S*A) reveal that a significantly larger fraction of nuclear 6/S*A (60.0±4.0%, at **p<0.005) was localized in the nucleoplasm as compared to that of WT (which is only at 35.5±8.5% of the total) (Figure S1). Occasionally we observe larger nucleoli, in cells expressing either NCL-6/S*A or WT. Such differences can be attributed to asynchrony of cell population and nucleolar fusion during S and G2 phase of the cell cycle that has been reported in the literature [44].

Because sub-nuclear translocation of NCL has been implicated in its role in regulating DNA replication, the cellular response to stress, and p53 activation, we examined NCL localization before and after genotoxic stress in inducible NCL cells. Both NCL variants (WT and 6/S*A) translocate completely to nucleoplasm upon treatment with the topoisomerase I inhibitor camptothecin (CPT, 2 µM for 2 h). Exposure to UV (50 J/m^2^) had a reduced effects on each variant, with WT and 6/S*A both showing a combination of nucleolar and nucleoplasmic localization (Figure 1E). These data indicate that the NCL-6/S*A mutation mimics the effect of stress by causing partial NCL translocation from the nucleolus to the nucleoplasm even under non-stress conditions.

These studies used a static approach to measure NCL localization. The greater nucleoplasmic localization of the 6/S*A mutant suggests a more mobile NCL. Therefore we investigated the effect of the mutation on NCL dynamics to measure its intra-nuclear mobility by fluorescence recovery after photobleaching (FRAP). FRAP experiments revealed that when nucleoli expressing GFP-tagged NCL were photobleached, ∼4 s faster recovery of fluorescence was observed with NCL phospho-mutant (*p<0.05, Figure S2). At least 10 data sets were analyzed by FRAP as described [45]. Although genotoxic stress caused greater mobility of both WT and the 6/S*A mutant, the mutant consistently showed higher mobility compared to WT under these conditions (*p<0.05, Figure S2).

To investigate the mechanism of nucleolin regulation by CK2, we performed *in vivo* phosphorylation assay in the presence of CK2 inhibitor DRB (5, 6-Dichloro-1-β-D-ribofuranosylbenzimidazole) and analyzed NCL phosphorylation as well as sub-nuclear localization. As indicated in Figure S3, we observed a significant decrease in ^32^P labeled NCL in the presence of CK2 inhibitor DRB when equivalent amount of NCL immunoprecipitates were assessed. Intriguingly, although use of the CK2 inhibitor DRB can be expected to have more pleiotropic effects, DRB treatment of cells also resulted in greater NCL mobilization (Figure S3).

These data strongly suggest that NCL hypophosphorylation at the consensus CK2 sites mobilizes NCL from the nucleoli in a manner similar to that earlier reported during cellular stresses (Figure 1E, [5], [7], [9]).

### Inducible expression of nucleolin phospho-variants activate the p53 checkpoint

**Figure 2:**
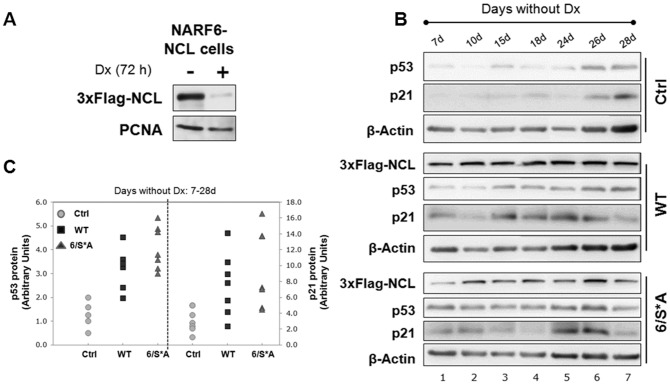
NARF6-NCL clones with inducible NCL (WT or 6/S*A) expression. **(A)** Western blot of a representative clone that expresses 3xFlag-NCL under Tet-off promoter in NARF6 cells (derived from U2OS). **(B)** Western blot analyses for cells grown without Dx for the indicated period showing inducible NCL-expression (WT or 6/S*A). Both WT and 6/S*A led to a net increase in p53 protein levels and corresponding p21 protein levels -the downstream target of p53. **(C)** Plots of p53 and p21 protein levels shown in 2B. The quantification was done by NIH Image J software. Values were first corrected for the β-actin levels and then compared to Ctrl (no exogenous NCL, no Dx day 7) cells. The graph is representative of at least three independent experiments.

We created retroviral constructs that express both the Tet activator and a 3xFlag-tagged NCL-WT or NCL-6/S*A from a single DNA molecule. We stably transfected NARF6 cells with these constructs; the NARF6 cells also express p14ARF from an IPTG-inducible promoter [41]. Stable clones were isolated that showed tetracycline (or doxycycline) regulated expression of NCL. Multiple clones were selected: Control cells (Ctrl, with no exogenous NCL expression, vector alone), WT (that express 3xFlag-NCL WT) and 6/S*A (expressing phosphorylation-deficient NCL mutant). Tests of a representative clone demonstrates expression of 3xFlag-NCL only upon doxycycline removal (Figure 2A) that almost completely shuts off when doxycycline is added back in the growth medium. In this study we present data from inducible NCL cells when exogenous NCL expression was induced by removal of doxycycline for a range of 1–28 days.

Earlier we reported that exogenous NCL expression stabilizes p53 levels and regulates its transcriptional activity [8], therefore, we examined the effects of NCL-WT and 6/S*A expression on p53 protein levels. When cells were induced continuously for WT and 6/S*A expression (from 7–28 days grown without doxycycline), both variants resulted in an increase in p53 protein levels although greater increase was observed with NCL-6/S*A expression (Figure 2B). Interestingly, NCL-WT expression showed dynamic expression (with periodic variation) of p53 levels when cells grown at different days without doxycycline. On the other hand, continuous induction of NCL-6/S*A expression resulted in more persistent (sustained) p53 protein levels. Corresponding to the p53 levels, increases in p21 protein-the downstream target of p53- were also observed (Figure 2B). The scatter plot representing the p53 and p21 protein levels during the 7 to 28 days of induced expression of WT or 6/S*A expression as compared to the Ctrl cells strongly indicated that both p53 and p21 levels were higher in 6/S*A expressing cells (Figure 2C). However, these mutant cells show fluctuating levels of p21 even with consistent p53 levels (Figure 2B, 2C). Control cells on the other hand had minimal effect on p53 or p21 levels during their growth without doxycycline.

We further characterized our NCL-expressing clones and confirmed that these cells have retained inducible p14ARF expression and subsequent p53 stabilization, as described earlier [41]. As depicted with two representative clones C1 and C2, both NCL-WT and p14ARF expression lead to an increase in p53 protein levels and a corresponding increase in p21 levels (Figure S4). Note that a smaller increase of p53 levels is observed with expression of NCL alone (Figure S4, lane 1 vs. lane 3). As expected, p53 half-life is drastically increased (beyond 2 h) upon robust p14ARF expression (Figure S5). Endogenous NCL half-life remains overall unaltered although a transient increase (of NCL) upon p14ARF expression is consistently observed (Figure S5; lanes 3, 4 vs. lane 2).

Next we tested the possibility of using these NCL expressing cell lines as an NCL-replacement tool. We utilized the fact that our 3xFlag-NCL constructs do not contain the 3′UTR (untranslated region) of the endogenous NCL mRNA and selectively downregulated the endogenous NCL by siRNA. Following two subsequent siRNA transfection and post 36 h of second siRNA treatment, we observe up to 70% reduction of endogenous NCL protein (anti-NCL blot, lower band). The induced NCL expression (i.e. the Flag-NCL by anti-NCL blot, upper band)) remained unchanged (Figure S6). In contrast siRNA that targets coding region of NCL gene downregulates both endogenous as well as induced NCL (data not shown, [8]). Although NCL silencing normally causes adverse effects on ribosome biogenesis [46], these novel cell lines allow us to replace the endogenous NCL with minimal toxic side effects when required. It should be noted that the induced NCL levels (at 10 d) are only 40% (for WT) or less (for mutant) that of endogenous NCL, we have therefore restricted endogenous NCL silencing to 60–70% to avoid any artifacts due to cell toxicity. The p53 protein levels were reduced with NCL-WT expression but remained unchanged or slightly increased with NCL-6/S*A expression, when endogenous NCL protein is reduced to 30–40%. Although we still observe some higher p21 levels upon endogenous NCL silencing as previously reported (Figure S6, [8]).

In summary, inducible NCL cells support the ability to express both p14ARF (in the presence of 1 mM IPTG, usually for 16–22 h) as well as full-length NCL phospho-variants (by removing doxycycline from growth medium for at least 24–48 h) in a very controlled manner. These are the first human cell lines that allow us to turn on or off the expression of two nucleolar proteins, p14ARF and NCL as well as can serve as an important NCL-replacement tool.

### Expression of nucleolin phosphorylation-deficient mutant increases p53 protein half-life

We focused on clones expressing different phospho-variants of NCL (WT or 6/S*A) to delineate the functional role/s of NCL phosphorylation by CK2 in regulating the p53 signaling pathway.

**Figure 3:**
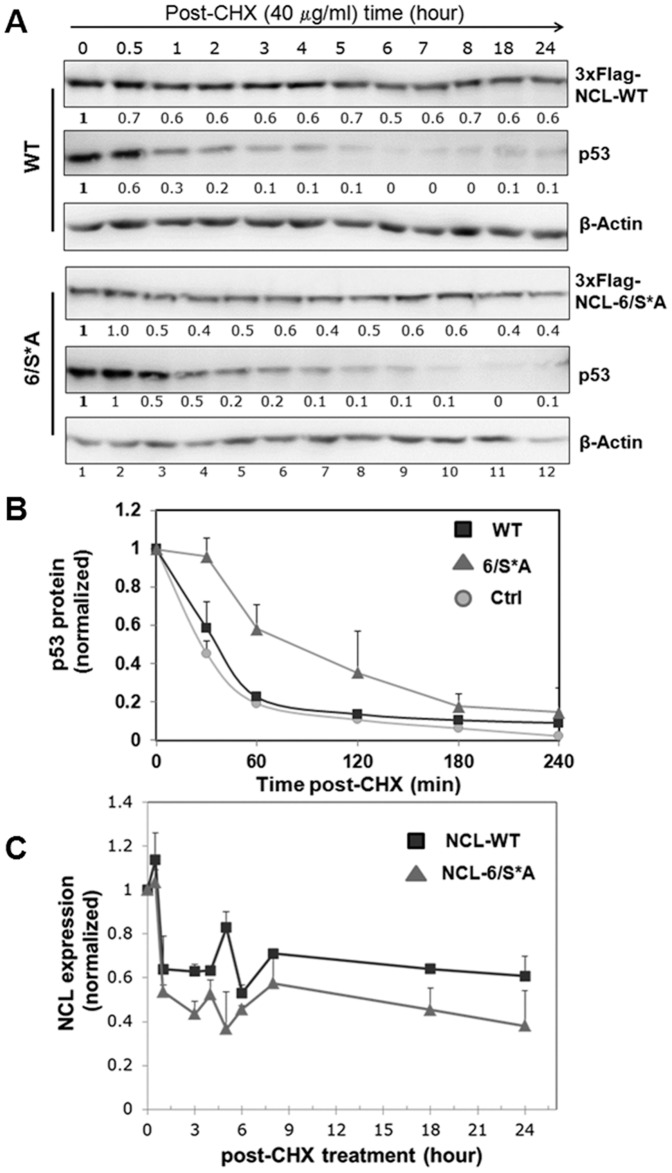
Half-life analyses of p53 and NCL (WT and 6/S*A) proteins. Inducible NCL-cells grown without Dx for 17d to express either NCL-WT or NCL-6/S*A. Ctrl represents control cells without exogenous NCL expression. Cells were then treated with cycloheximide (CHX, 40 µg/ml) for indicated times. **(A)** Lysates were prepared and analyzed by Western blotting for p53, FLAG (for NCL expression) and the β-actin loading control. The relative band intensities for NCL and p53 proteins were quantified following normalization with β-actin and are indicated below each blot. **(B)** Plot of p53-expression levels following CHX treatment corrected for the β-actin levels. The graph is representative of three independent experiments done in duplicates. Half-life of p53 is ∼60 min for NCL-6/S*A, ∼30–40 min for NCL-WT and ∼15–20 min for Ctrl (vector) expressing cells. **(C)** NCL-6/S*A expression levels are relatively low as compared to NCL-WT under steady state conditions. Half-life of NCL-6/S*A is significantly lower <2 h as against>6 h for NCL-WT suggesting CK2 phosphorylation might regulate NCL protein stability.

First, we determined the p53 protein half-life upon expression of NCL phospho-variants by inhibiting protein synthesis by cycloheximide. The p53 protein half-life was clearly increased with NCL-6/S*A expression as compared to WT as indicated by a representative Western blot (Figure 3A). The graph is a representative of three independent experiments, each performed in duplicate (Figure 3B). We further evaluated p53-stability for shorter time period following cycloheximide blocking. As indicated in Figure S7 (upper panel) the p53 half-life is lower in cells expressing WT (∼30–40') as compared to mutant (∼1 h), while control cells have normal half-life of ∼15–20'. Assuming the decrease in p53 protein levels is a pseudo-first order kinetic process, the data presented in Figure 3B were also plotted on a log scale to indicate indeed a higher p53 protein half-life in cells with mutant-NCL expression (Figure S7, lower panel). Interestingly, we also observed lower steady state levels of NCL-6/S*A which had a reduced half-life (<2 h) as compared to WT (>6 h; Figure 3C). The shorter half-life of NCL-6/S*A indicates that CK2 phosphorylation might regulate NCL protein stability as previously suggested by others [47], [48]. The observed fluctuations in the expression pattern for 6/S*A, in part is due to differential stability of nucleolar vs. nucleoplasmic levels of the protein [49].

### Nucleolin mutant, defective in CK2 phosphorylation, causes dominant-negative effect on cell proliferation in a p53-dependent manner

**Figure 4:**
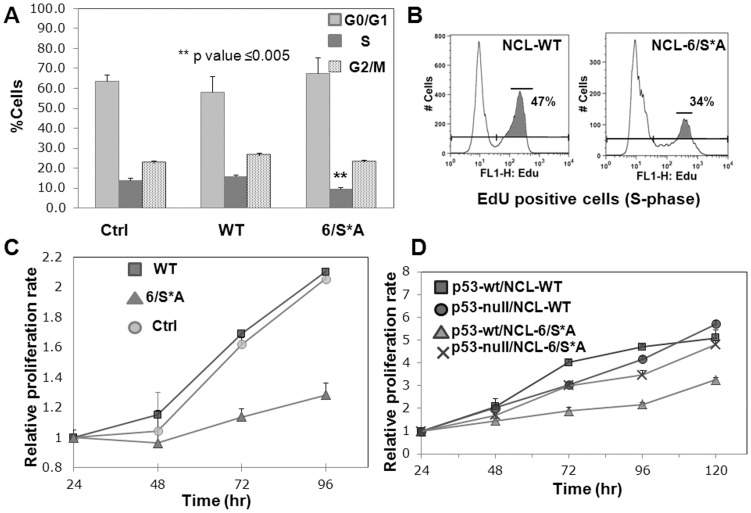
NCL-6/S*A expression causes p53-dependent inhibition of cell proliferation. **(A)** Ctrl (no exogenous NCL) and inducible NCL cells (WT or 6/S*A, induced for 6–8 d and 28 d) were analyzed for the DNA content by propidium iodide staining and flowcytometry. NCL-6/S*A expressing cells have significant low % of cells in the S-phase as compared to WT expressing or Ctrl cells (**p value ≤0.005). **(B)** Reduced % of cells in S-phase with 6/S*A expression for 10 d as assayed by Click-iT EdU flow cytometry kit (Invitrogen). Analyses were performed using FlowJo 9 software. The data is representative of three independent experiments performed with inducible NCL cells (WT or 6/S*A, induced for 10–20 d). **(C)** Continuous expression of 6/S*A (17 d) is inhibitory to cell-proliferation as analyzed by MTS assay. The data is representative of at least three independent experiments performed with Ctrl, WT or 6/S*A (inducible NCL) cells. Each point represents the mean ± SD of 6–8 replicates. **(D)** Inhibition of proliferation by NCL-6/S*A expression requires p53. HCT116-p53 wt or null cells were transfected with NCL-WT or -6/S*A, and assayed for cell proliferation using MTS solution. Each point represents the mean ± SD of 6–8 replicates.

To understand the physiological consequences in terms of cellular fate upon the stabilized p53 protein levels, we examined cell cycle progression and cell proliferation upon induced NCL-WT and 6/S*A expression. Measurement of DNA content by propidium iodide staining and subsequent profiling of the cell cycle distribution revealed a significant decrease in the fraction of cells in S-phase upon NCL-6/S*A expression (Figure 4A, **p value ≤0.005). We also employed a more sensitive method to determine to S-phase population, using a Click-iT EdU flow cytometry assay to directly measure DNA synthesis. This assay incorporates EdU (5-ethynyl-2′-deoxyuridine), a thymidine analog into DNA during an active DNA synthesis. As indicated in Figure 4B, a representative of three independent experiments, EdU positive cells were reduced from 47% (in WT) to 34% in 6/S*A expressing cells.

The decreased proportion of cells in S-phase strongly suggest that cells with continuous expression of NCL-6/S*A delay the cell cycle, failing to progress through the S-phase. Indeed the MTS cell proliferation assays demonstrated that NCL-6/S*A was inhibitory to cell proliferation as compared with WT expressing or Ctrl cells (Figure 4C).

To determine if the NCL-6/S*A mediated inhibition of cell proliferation is p53-dependent, we used HCT116 p53-wt and null cells [50]. As depicted in figure 4D, there was no significant difference in proliferation rate with expression of NCL (WT or 6/S*A) in p53-null background although lesser viability is evident with 6/S*A. In the presence of p53-wt however, NCL-6/S*A expression showed a significantly lower rate of cell proliferation as compared to NCL-WT (Figure 4D). These data decisively demonstrate that NCL phosphorylation-deficient mutant inhibits cell proliferation in a p53-dependent manner. Intriguingly, the inhibitory effect by NCL-6/S*A occurs in the presence of endogenous NCL-WT. Thus, expression of phosphorylation-deficient NCL mutant exerts dominant-negative effect on cell proliferation.

All the three cell lines (Ctrl, expressing either inducible NCL-WT or NCL-6/S*A) showed a comparable standard curves showing linear 490 nm absorbance pattern to the number of living cells (Figure S8, upper panel). As anticipated, all cells, in the absence of induced NCL expression (i.e. when grown in presence of doxycycline) had similar proliferation pattern (Figure S8, lower panel).

### Nucleolin (WT and 6/S*A) associate with endogenous-NCL

**Figure 5:**
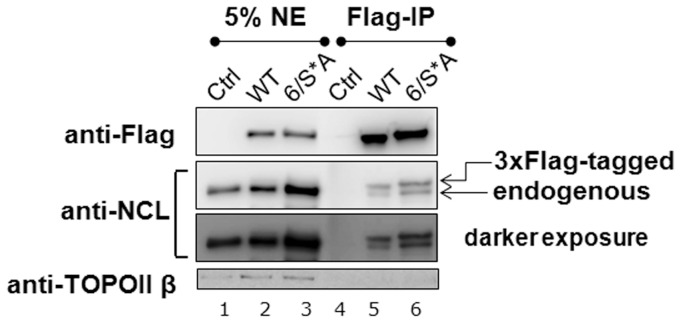
NCL-WT and 6/S*A interact with endogenous NCL. Nuclear extracts (NE) were prepared from cells grown without Dx for at least 10 d for NCL-WT or 6/S*A expression. Ctrl represents control cells without exogenous NCL expression. Equal amounts of NE protein from these cells were then subjected to co-immunoprecipitation using anti-FLAG M2 beads. Western analyses of NE and bound fractions were analyzed by anti-NCL (to detect: -exogenous 3xFlag-tagged NCL, upper band and –endogenous NCL, lower band), anti-Flag, anti-p53 and anti-p21. Anti-TOPOII β blot serves as the loading control for NE. The data is representative of three independent experiments performed with 10 d–20 d of WT or 6/S*A expression. This data supports the hypothesis that NCL (both WT and 6/S*A) can associate with endogenous NCL (i.e. NCL-NCL interactions).

We observe a dominant-negative effect on cell proliferation by NCL-6/S*A. Earlier it was shown that NCL oligomerizes through its RNA binding domain [40]. This leads to our hypothesis that NCL-6/S*A binds to NCL-WT and negates WT functions. To test our hypothesis, we performed co-immunoprecipitation assays to precipitate Flag-tagged inducible NCL by M2-beads and analyzed the bound fraction for endogenous NCL. Indeed endogenous NCL was precipitated along with both Flag-tagged NCL-WT and 6/S*A (Figure 5). These data demonstrate that NCL-6/S*A can antagonize NCL-WT through protein-protein interactions.

### Lack of nucleolin phosphorylation by CK2 activates expression of apoptotic markers downstream in the p53 pathway

**Figure 6:**
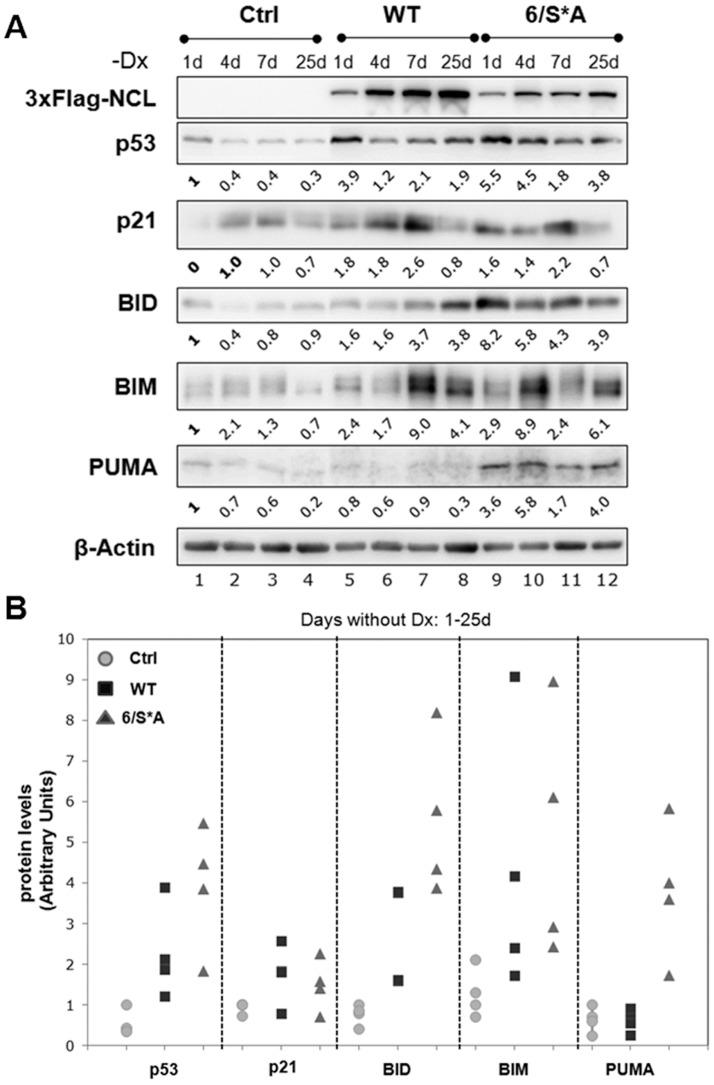
NCL-6/S*A expression results in an increased expression of apoptotic markers downstream the p53 pathway. **(A)** Western blot analyses for cells grown without Dx for the indicated period indicating inducible NCL-expression (WT or 6/S*A). Both WT and 6/S*A expression resulted in an increase in p53 and p21 protein levels. Increased expression of BH3-only pro-apoptotic markers (BID, BIM and PUMA) were observed as early as 1 d or 4 d of induced NCL-6/S*A expression. **(B)** Plots of p53, p21 and BH3-only protein levels shown in 6A. The quantification was done by NIH Image J software. Values were first corrected for the β-actin levels and then compared to Ctrl (no exogenous NCL, no Dx day 1) cells. The graph is representative of at least two independent experiments.

To further elucidate NCL-6/S*A mediated inhibition of cell proliferation, we analyzed markers in apoptotic pathway to obtain information about the observed cellular fate. Since continuous NCL expression causes activation of the p53 checkpoint (Figure 2B), we examined cells with shorter (1 d, 4 d, 7 d) and longer (25 d) NCL induction times to obtain a functional understanding of this activation. As depicted in Figure 6A, we observe increases in p53 levels with both WT and 6/S*A expression as early as 24 h of NCL induction. We also saw that p53 levels were fluctuating even at early time points of NCL-WT induction (e.g. 1 d, 4 d) as compared to 6/S*A expression (Figure 6A, compare lanes 5,6 vs. 9,10). As mentioned earlier, p21 levels were mostly comparable to corresponding p53 levels. Induced expression of NCL-6/S*A resulted in an increased level Bcl-2 homology 3 (BH3)-only apoptotic marker proteins, BID (BH3 interacting-domain death agonist), BIM and PUMA (p53 upregulated modulator of apoptosis) as compared to the Ctrl or NCL-WT expressing cells (Figure 6A). Interestingly, these apoptotic markers are also expressed as early as post 24 h of inducible NCL mutant expression. The scatter plot presented in Figure 6B clearly indicates that NCL-6/S*A expression causes higher levels of BH3 only proteins, especially BID and PUMA, suggesting a role for regulation of apoptosis in these cells.

We observed a similar increase in p53, BID and PUMA with NCL mutant expression for longer induction periods (10–24 d, Figure S9). A cumulative scatter plot for all the combined data (with as little as 24 h to as long as 28 days of induced NCL expression) clearly reveals increased levels of p53, BID and PUMA with NCL-6/S*A expression as compared to WT or Ctrl cells (Figure S10). The increases in p21 protein levels were not significantly different over the tested induction period between WT and 6/S*A.

The BH3-only proteins have been strongly implicated in triggering Bax/Bak mediated apoptosis either directly or indirectly by targeting the pro-survival proteins [51], [52]. BID and PUMA are transcriptional targets of p53, however, PUMA is essential for p53-independent apoptotic pathways as well. BIM on the other hand is not a direct p53 target but is required during DNA damage induced cell death [53]–[55]. Although an initial increase in BIM levels were observed in 6/S*A expressing cells especially at early times in NCL induction (Figure 6, for 1 d, 4 d), BIM levels were not significantly different among the Ctrl, WT and 6/S*A cells, at later time points. However, there is a possibility that lack of NCL phosphorylation by CK2 triggers BIM expression early on in response to cellular stress that described with sub-nuclear mobilization earlier. Together, limiting NCL phosphorylation by CK2 can initiate apoptosis through both p53-dependent and independent mechanisms.

## Discussion

**Figure 7:**
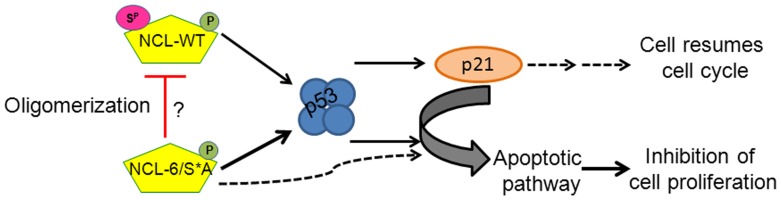
Mechanistic model by which nucleolin phosphorylation by CK2 regulates cell proliferation. NCL-WT and phosphorylation-deficient mutant (6/S*A) activate p53 checkpoint and increase corresponding p21 levels. However, NCL-WT expression allows cells through S-phase progression and resumes cell cycle. On the other hand, NCL-6/S*A acts as a dominant-negative mutant that negates NCL-WT functions possibly through oligomerization causing inhibition of cell proliferation and initiating apoptotic pathway.

In the current study we have generated new cellular tools that express CK2-specific full-length NCL phosphorylation variants upon a Tet-off inducible promoter system. Using this approach, we make the novel observation that NCL phosphorylation by CK2 at the six consensus sites is required for cell survival and cell proliferation. Loss of phosphorylation at these CK2 sites results in increased p53 levels activating the signaling pathway downstream to p53. Expression of phosphorylation-deficient NCL mutant causes a reduced fraction of cells in S-phase that ultimately leads to an inhibition of cell proliferation presumably by initiating apoptosis pathway (Figure 7). In contrast, NCL-WT expression causes pulsatile p53 and p21 protein levels that allow cells to survive probably by resuming the cell cycle progression.

In recent years, modulation of the stress response (via activated p53 checkpoint) by nucleolar factors has been recognized to play an important role in tumorigenesis [56]–[60]. NCL, the major nucleolar protein regulates p53-signaling at multiple levels: in exponentially dividing cells, heightened NCL levels can trigger a p53-checkpoint by NCL binding to Hdm2 and reducing p53 ubiquitination [8], [9]. This may reflect a feedback mechanism that serves to prevent hyperproliferation induced by abnormally high NCL levels. During the DNA damage response, NCL can translocate from its usual nucleolar locations to nucleoplasm where it can modulate the translation of p53 through NCL-RPL26 or NCL-NCL oligomerization [40] and/or regulate p53 protein through direct interactions with p53 [5], [7], [9] or its antagonist Hdm2 [8], [9].

In this study we extend the regulation of p53 by NCL phosphorylation demonstrating that dephosphorylation at CK2 sites leads to “nucleolar stress” like conditions that allow NCL to mobilize and activate the p53 signaling pathway. In fact, both WT and 6/S*A expression increase the p53 protein levels from day1 and we observe an increased p53 protein stability associated with NCL-mutant expression. We frequently observe more dynamic p53 levels with NCL-WT expression while p53 levels persist with continuous NCL mutant expression. Remarkably, only induced NCL-6/S*A expression (even in the presence of the endogenous NCL) results in a dominant-negative effect on cell proliferation that is predominantly p53-mediated. We envision that NCL-6/S*A negates the effects of endogenous NCL through oligomerization and indeed we observe interactions between endogenous-NCL and exogenous-NCL (WT or 6/S*A) in our co-IP assays to support this concept. Future stoichiometric analyses between nucleolin and its different phosphorylation forms will provide more insights about nucleolin oligomerization and its physiological implications. In contrast, WT cells survive and proliferate through fluctuating p53 levels. Recently from single cell analyses, the phenomenon of pulsed versus sustained p53 levels and different cellular fate has provided useful insights about p53 control of gene expression in DNA repair, apoptosis and senescence pathways [61]. Expression of phosphorylation-deficient NCL results in consistent p53 levels and activates pro-apoptotic proteins e.g. BID and PUMA. These BH3-only proteins expression is known to be transcriptionally activated upon DNA-damaging chemotherapeutic agents to activate the destructive caspase cascade resulting in cell death. Although our data strongly support that NCL mutant requires p53-mediated signaling in restricting cell proliferation, a p53-independent increase of PUMA and BIM cannot be completely ruled out. It is also possible that targeting NCL phosphorylation by CK2 could synergistically initiate a broader range of BH3-only proteins for more efficient retardation of proliferation.

Together, hypophosphorylated NCL with more nucleoplasmic localization presumably causes “nucleolar stress” like conditions that can trigger multiple events: NCL binds to p53-protein and RNA directly or modulates p53 levels through interactions with other p53-regulators (e.g. Hdm2 or RPL26); other nucleolar factors can similarly sense this stress to regulate p53 checkpoint [62]. Thus, increases in p53-levels in NCL-mutant expressing cells could be the net result of these direct and indirect events.

Occasionally larger nucleoli associated with inducible NCL expression could reflect its functional capability; more nucleolar specific RNA cytochemistry analyses are warranted to identify a role for NCL hypophosphorylation in these processes. Future studies with single cell analyses will prove highly useful in validating the functional impacts of NCL hypophosphorylation in regulating the kinetics of the p53 signaling pathway.

It is worth mentioning that NCL is also an important RNA binding protein that regulates gene expression through direct NCL/RNA interactions or indirectly by recruiting other molecules in RNA metabolism. Importantly, the N-terminus and the RNA binding domains of NCL are positioned adjacently and hence can influence NCL protein conformation and/or functional properties [47]. In spite of this association, due to the highly-acidic nature of the N-terminus and technical issues of purifying full-length NCL, the role of NCL phosphorylation has been overlooked while studying many of its physiological functions. These NCL constructs and clones are therefore valuable in facilitating the full-length NCL purification through its tag (His, Flag). Additionally, these cells have an ability to express p14ARF tumor suppressor protein that lies upstream to the p53 checkpoint activation, more commonly during oncogenic stimulation. Using the inducible expression of NCL in this study we reveal for the first time how CK2-mediated NCL phosphorylation, provides necessary quality control surveillance on the major cellular decision of cell survival.

## Material and Methods

### Cell culture and reagents

U2OS (osteosarcoma, p53-wt, ARF-null) cells were obtained from ATCC (American Type Cell Culture). NARF cells [41] were kindly provided by Dr. Gordon Peters. All cell lines were grown in DMEM containing 10% FBS and 100 units penicillin-streptomycin, at 37°C with 5% CO_2_ atmosphere in a humidified incubator. Plasmid transfections were performed using Effectene transfection reagent (Qiagen). SIGMAFAST protease inhibitor cocktail tablets (Sigma-Aldrich, St Louis, MO, USA) were used at 1× concentrations as per the manufacturer's instructions. For various treatments, the drugs were directly added to the growth media to a final concentration as indicated: Isopropyl β-D-1-thiogalactopyranoside (IPTG, Sigma-Aldrich) at 1 mM; camptothecin (CPT, Sigma-Aldrich; a stock concentration of 10 mM in DMSO) at 2 µM; 5, 6-Dichloro-1-β-D-ribofuranosylbenzimidazole, DRB (a CK2 inhibitor, Sigma- Aldrich) at 40-60 µM and cycloheximide (CHX, Sigma- Aldrich) at 40 µg/ml for indicated time periods. For UV treatment, cultures at 90% confluency were exposed to UV dose (50 J m^−2^) using a UVP HL-2000 HybriLinker (with a 254 nm UV cross linker from Fisher Scientific). Before pulsing, the medium was removed and same medium was replaced immediately after treatment. All reagents were of molecular or cell culture grade and were obtained from Fisher Scientific unless otherwise mentioned.

### Antibodies

The primary antibodies used for Western blotting were as follows: GFP, rabbit polyclonal (Molecular Probes, Invitrogen Corp. Carlsbad, CA, USA); FLAG, M2-monoclonal or rabbit polyclonal and β-actin, mouse monoclonal (all from Sigma-Aldrich, St Louis, MO, USA); p14ARF (14P02, rabbit polyclonal, Thermo Fisher Scientific Biosciences); NCL, mouse monoclonal MS-3 and rabbit polyclonal H250, NPM (B23, 0412), mouse monoclonal, p53 mouse monoclonal DO-1, PCNA, rabbit polyclonal and TOPO II β, mouse monoclonal (all from Santa Cruz Biotechnology, Santa Cruz, CA, USA). Pro-apoptotic Bcl2 family antibody kit and anti-p21, mouse monoclonal were purchased from Cell Signaling technology. The secondary antibodies used were anti-mouse and anti-rabbit HRP-conjugated antibodies (GE Healthcare Bio-Sciences Corp. Piscataway, NJ, USA). Various reagents for protein purification and biochemical assays were purchased commercially including Ni^+2^-NTA agarose beads (Qiagen, Valencia, CA, USA) and FLAG M2 beads (Sigma-Aldrich, St Louis, MO, USA).

### NCL mutagenesis and retroviral infection

The expression constructs for human nucleolin (NCL, NCBI: NP_005372.2 or UniProt: P19338); full-length (FL, aa 1-710) containing an N-terminal GFP-tag was described previously [7], [8]. Earlier we have generated NCL phospho-mutants with three putative CK2 sites at positions S34, S184, and S206 converted to non-phosphorylatable alanine [3/S*A, earlier designated as NCL-TM, triple mutant [7]]. Subsequently, we mutated three additional CK2 phosphorylation sites to further lower phosphorylation by CK2 [24], and generated a novel reagent 6/S*A (six serine mutated to alanine: S28A, S34A, S145A, S153A, S184A, S206A). Sequential site-directed mutagenesis for NCL phospho-mutants was done using the QuikChange site-directed mutagenesis kit (Stratagene). Top strand primer sequences used were:

S28A

5′ TCCAAAGGAGGTAGAAGAAGATGCTGAAGATGAGGAAATGGC 3′

S145A

5′ ATGCCGAGGAGGAAGACGCTGATGAAGAGGAGGATG 3′

S153A

5′ AAGAGGAGGATGATGACGCTGAGGAGGATGAGGAGG 3′

Constructs with different tags (e.g. His, FLAG and GFP) were created for NCL (-WT or 6/S*A). PCR reactions (for WT and 6/S*A constructs) were performed using 3xFlag-tagged NCL as a template and generated full-length NCL coding sequence with the primers that contain Not I and BamH I sites in the forward and reverse primers respectively. PCR products were subcloned into the Not I/BamH I sites on the pRetro-Off retroviral vector (Clontech). Production of retroviruses containing the NCL (WT or 6/S*A) expression cassettes were performed in Phoenix cells (Retroviral Helper dependent protocol, https://www.stanford.edu/group/nolan/protocols/pro_helper_dep.html). Subsequently, NARF6 cells were infected with retroviral constructs (NCL-WT or 6/S*A). Stable clones that allow inducible expression of either 3xFlag tagged NCL-WT or -6/S*A driven by a Tet-Off inducible promoter were isolated and expanded as described elsewhere [64]. Multiple clones that can turn on NCL expression by removal of doxycycline (Dx, semi-synthetic tetracycline) from the culture medium were selected. Addition of doxycycline in contrast, shuts off exogenous NCL expression ∼90% in these selected clones. These NCL-clones were grown without doxycycline in the medium for the indicated time (hours or days) for NCL-expression and used in subsequent biological and biochemical assays as described. In each selective assay, analyses of multiple days of NCL-induction were included to obtain a representative outcome and possible insights into dose-dependent response.

### Purification of NCL phospho-variants

His-tagged NCL proteins were purified on Ni^2+^-NTA Agarose beads (Qiagen), using the manufacturer's protocol. Briefly, U2OS cells were transfected with constructs expressing His-tagged NCL (WT or 6/S*A). At 48 h post-transfection, cells were lysed in buffer containing 50 mM NaPO_4_ pH 7.4, 1% (v/v) NP-40, 0.05% (v/v) Tween 20, 0.5 M NaCl, 20% (v/v) glycerol, 20 mM imidazole, 0.5 mM phenylmethylsulfonyl fluoride (PMSF), 2 mM sodium vanadate (Na_3_VO_4_), 50 mM NaF, 1 mM dithiothreitol (DTT) and benzonase. His-tagged proteins were purified using Ni^2+^-NTA agarose beads by incubating for 2 h at 4°C on a shaker. Beads were washed 5 times with wash buffer [50 mM NaPO_4_ pH 7.4, 200 mM NaCl, 20% (v/v) glycerol, 20 mM imidazole, 0.5 mM PMSF, 2 mM Na_3_VO_4_, 50 mM NaF, and 1 mM DTT]. Proteins were then eluted with elution buffer (50 mM NaPO4, pH 7.4, 300 mM imidazole, and 20% glycerol). Afterwards, the eluate was dialyzed overnight at 4°C against phosphate-buffered saline (PBS) and 20% glycerol in the presence of protease inhibitors. Purified proteins in the eluate were assayed for purity by SDS-polyacrylamide gel electrophoresis (PAGE) and Coomassie blue staining.

### Phosphorylation assay

To assess NCL phosphorylation, the NCL variants were purified as described above and were analyzed by SDS-PAGE, using two 6% polyacrylamide gels. One gel was fixed in a solution containing 50% methanol and 10% acetic acid, and first stained using Pro-Q Diamond phosphoprotein gel stain (Molecular Probes) in accordance with the manufacturer's instructions. The Pro-Q Diamond staining specific for phosphorylated protein was visualized on a Typhoon Trio scanner (GE Healthcare) using excitation at 532 nm florescence and 555 BP 20 filter to record emission. The same gel was subsequently stained with SYPRO Ruby (Molecular Probes) for total protein analysis and emission recorded using 610 BP 30 filter. The second parallel gel was transferred onto a nitrocellulose membrane for Western analyses and was probed for anti-NCL. Intensities of bands corresponding to phosphorylated nucleolin (from Pro-Q staining) and total nucleolin (from SYPRO staining) were determined by Image J analysis. Relative nucleolin phosphorylation ratio was then calculated for the Pro-Q signals of NCL-6/S*A to that of NCL-WT, after normalizing for the amount of total nucleolin.

### NCL phosphorylation *in vivo*

U2OS cells were transfected with GFP-tagged NCL-WT and labeled metabolically with ^32^PO_4_ for 90 minutes. CK2 inhibitor, DRB was used at 40 µM for 24 h as indicated. We performed immunoprecipitation (IP) assay to pull-down tagged-NCL. IP fractions were subjected to SDS-PAGE and the relative amount of total ^32^PO_4_ incorporation in NCL-WT was assayed by autoradiography. IP fractions were also detected using anti-NCL antibodies on a parallel Western blot to confirm equal amount of immunoprecipitates in each sample. Simultaneously, sub-nuclear localization of GFP-tagged NCL-WT in the presence or absence of DRB was recorded using an epifluorescence microscope (Olympus IX71).

### Immunofluorescence staining

Immunofluorescence technique was employed essentially as described earlier [8]. Briefly, cells were first split onto coverslips, either transfected or induced for NCL expression (15–29 d of Dx removal). Post-36h of transfection or indicated time point following induction cells were either mock- or CPT-treated. For treatment, cells were incubated with camptothecin (CPT, 2 µM for 2 h) or exposed to UV dose (50 J m^−2^) and incubated post-UV for 25 min. Cells were then washed with PBS and fixed with 4% (w/v) paraformaldehyde. Next, cells were permeabilized with 0.5% (v/v) Triton X-100 in PBS for 5 min at RT. After washing twice with PBS, coverslips were then either directly viewed using FITC filter (for GFP-NCL expression) or incubated with rabbit anti-Flag primary antibodies (for 3xFlag-NCL) followed by Alexa-594 labeled anti-mouse secondary antibodies (Molecular Probes Inc.). Image acquisition was performed using Olympus IX71 inverted epifluorescence and Olympus FV10i confocal scope (Tokyo, Japan).

Semi-quantitation of nucleolar vs. nucleoplasmic staining was performed using Metamorph advanced analyses program (Molecular Devices, Sunnyvale, CA). Briefly, each nucleus following nucleolin immunostaining was photomicrographed at the constant light level with a 40× objective. Each photomicrograph was taken consecutively using Texas Red and DAPI filter sets (Chroma, Bellow Falls, VT). For each image, using the threshold above the background, total number of nuclei were selected and then quantified using MetaMorph's Integrated Morphometry Analysis (IMA) feature. Using DAPI mask, first the total number of nuclei were identified and average integrated intensities of each nucleus was measured using Texas Red filter. Similarly, by copying few nuclei regions (outlines) into the non-cell areas of the image, background intensities were measured. The average of background intensities were subtracted from the total nuclear measured intensities. Next, by changing the threshold value integrated intensities of all the nucleoli were measured similarly in the red channel. Nucleoplasmic staining intensity was obtained by subtracting the total nucleolar staining from the nuclear staining and % staining for each sub-nuclear location (nucleolar and nucleoplasmic) were calculated. X/Y location parameters were used to identify each nucleus and its corresponding nucleoli. The size and area filters were employed to minimize intrinsic variability in size of the nuclei. To quantitate the sub-nuclear distribution of NCL localization, we examined a total of ∼80–100 nuclei with differential levels of NCL expression (low, medium and high) for both WT and 6/S*A. Sub-nuclear distribution of NCL in cells (n = 30 for each WT and 6/S*A) with moderate level of NCL expression as determined by the integrated morphometric analyses were used to achieve statistical analyses.

### FRAP analysis

U2OS cells were grown and transfected with the appropriate GFP-tagged expression constructs (NCL-WT or 6/S*A) in 35-mm uncoated glass bottom cell culture dishes (MatTek). Twenty-four hours post-transfection, cells were either untreated or treated with CPT as described above. Under our conditions, U2OS cells contain an average of 4 to 8 nucleoli per cell. Live cell images were obtained with a Zeiss LSM510 Meta laser scanning confocal microscope with a Plan-Apochromat 63× objective (1.4 NA) and a 30-mW Argon laser set at 50% of total output. Prior to photobleaching, five images were taken of the nucleus containing the nucleolar region of interest (ROI). One nucleolus per cell was therefore chosen as an ROI and bleached using the 488 nm laser line. After photobleaching of the ROI, images were then acquired every 5 seconds for at least 60 seconds, when fluorescence had recovered to maximal intensity for at least 15 sec. At least 10 datasets were analyzed for each result. Fluorescence intensity was measured using Zeiss image-processing software. The average intensity in the ROI before bleaching, immediately after bleaching and post bleaching was measured. Fluorescence intensity of the nucleus was also measured. Background fluorescence was measured in a field outside the cell and subtracted from the nucleolar and nuclear fluorescence values. The relative fluorescence intensity (RFI) was calculated as follows [45]: 
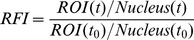


Where ROI (t) is the average fluorescence intensity of the photobleached region at various time points after photobleaching, Nucleus (t) is the average fluorescence intensity of the entire nucleus at the corresponding time point, ROI (t_0_) is the average fluorescence intensity of the photobleached region before photobleaching (time zero), and Nucleus (t_0_) is the average fluorescence intensity of the entire nucleus before photobleaching. Non-linear curve fitting of the recovery data was carried out using ORIGIN (Microcal Software). The fluorescence recovery (F) was plotted against time (t) and t1/2 was calculated using the equation: 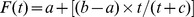 where ‘a’ is the intensity value immediately after bleaching, ‘b’ is the maximum intensity value and ‘c’ is t1/2.

### Nucleolin replacement strategy and silencing

For replacement of endogenous NCL, NCL-expressing clones were first grown for the indicated period in medium lacking doxycycline to allow ectopic NCL expression. The endogenous NCL was then downmodulated using an siRNA (purchased from Dharmacon, Inc, Thermo Fisher Scientific Biosciences) molecule that targets the 3′UTR of NCL gene using siNCL-A4 Target sequence: GAGUUGAGUGAUAGAGCUAUU. In contrast, siNCL-A2 Target sequence GCAAAGAAGGUGGUCGUUU targets coding region of NCL gene and hence affects both endogenous as well as exogenous NCL (i.e. the Flag-tagged NCL). Silencing was achieved using RNAiMax (Invitrogen) as per the manufacturer's instructions. Two consecutive siRNA treatments were performed to achieve higher level of NCL downregulation. Cells were tested 72 h post-transfection (∼36–40 h post 2^nd^ siRNA treatment). siGENOME non-targeting siRNA2 (Dharmacon, Inc, Thermo Fisher Scientific Biosciences) was used as control siRNA (Ctrlsi).

### Nuclear extract preparation and co-immunoprecipitation assay

NEs were prepared from harvested cells using standard protocols. Briefly, cells were lysed by douncing in 4 ml of hypotonic buffer [10 mM Tris pH 8.0, 1.5 mM MgCl_2_, 10 mM KCl, 1× protease inhibitor cocktail and 0.5 mM PMSF]. Lysates were centrifuged for 10 min at 6000 g, and pellets were resuspended in nuclear extraction buffer (50 mM Tris pH 8.0, 150 mM NaCl, 1.0 mM EDTA, 1% Triton X-100, 1x protease inhibitor cocktail and 0.5 mM PMSF). Preparations were rocked for 30 min at 4°C and centrifuged for 15 min at 10 000 g. Supernatants were quick frozen and stored at −80°C. 10% of NE was saved and ∼100–200 µg of total protein from NE were immunoprecipitated using EZview red anti-FLAG M2 Affinity Gel (Sigma) to pull-down Flag-tagged NCL following manufacturers' recommendations. Protein estimation is performed by Coomassie Plus (Bradford) Protein Assay (Thermo Fisher Scientific Biosciences).

### Western blot assay

Cells were directly lysed in 2×SDS dye and separated by SDS-PAGE. Proteins were then immobilized onto nitrocellulose membranes (0.2 µm pore size). After blocking in non-fat dry milk (0.5%, w/v) for 1 h at RT, the membrane was incubated with primary antibodies at 4°C overnight, HRP-conjugated anti-mouse IgG or anti-rabbit IgG was used as secondary antibody. The membranes were visualized by ECL-plus reagent (Perkin-Elmer, Wellesley, MA, USA), scanned and analyzed using ImageQuant LAS 4000 biomolecular imager (GE Healthcare Bio-Sciences AB, Sweden). NIH Image J software was used to quantitate band intensities of Western blots. Values were first corrected for the corresponding loading control and the normalized value of a particular marker as against the experimental control is then presented below each blot, as indicated. Lighter intensity blots were used in analyses by Image J. Darker exposure blots are also provided, when required. Multiple blots were used to quantitate various markers and different samples. Spliced out and combined lanes are clearly indicated with the vertical lines in the figures.

### Protein half-life assay

Cells were continuously grown in DMEM medium without Dx to induce expression of 3xFlag-tagged NCL-WT or -6/S*A for indicated period. Protein synthesis was inhibited by incubating cells with 40 µg/ml cycloheximide (CHX). Cells were lysed with 2×SDS-PAGE dye at various times post-CHX incubation as indicated. Lysates were then analyzed by Western blotting for p53, FLAG (for NCL expression) and the β-actin loading control.

### Flow Cytometry Analyses

Cells were trypsinized by 0.25% Trypsin-EDTA solution and then washed with PBS twice. Cells were fixed by drop-wise addition into ice-cold 70% ethanol and incubated on ice for 30 min. Cells were then stained with 0.5 ml of a solution containing PBS, 100 µg/ml propidium iodide (Sigma), 0.1% (v/v) Triton X-100, and 200 µg/ml RNase A, overnight at 4°C. Cells were analyzed next day by a BD Accuri C6 flow cytometer (BD Biosciences). For detection of cells specifically in the S-phase, Click-iT EdU Alexa Fluor 488 flow cytometry kit (Invitrogen) was used as per manufacturer's instructions. We used Becton-Dickinson FACSort to measure the fraction of cells in S-phase using CellQuest Pro software (BD Biosciences). Flow data were then analyzed using FlowJo 9 and positive EdU signal is indicated as the S-phase cell population.

### Cell proliferation assays

MTS [3-(4, 5-dimethylthiazol-2-yl)-5-(3-carboxymethoxyphenyl)-2-(4-sulfophenyl)-2H-tetrazolium, inner salt] assay was used to determine cell proliferation rate. Cells were split at 5000/well into 96-well plates, and harvested at 24 h, 48 h, 72 h, and 96 h. Following harvesting, CellTiter 96 AQueous One Solution Cell Proliferation Assay (MTS) solution (Promega) was added at 20 µl/well and incubated 4 h at 37°C. OD values were measured at 490 nm by BioTek plate reader.

## Supporting Information

Figure S1 Sub-nuclear distribution of NCL (WT and 6/S*A). Inducible NCL cells grown without doxycycline for 15–29d were used to detect NCL by immunofluorescence using anti-Flag antibodies. Image acquisition was done with constant parameters between the samples. Integrated Morphometry Analysis (IMA) was performed for ∼80–100 nuclei as described. Upper panel is representative image at moderate level of NCL expression. The graph represents sub-nuclear distribution of moderately expressed NCL (WT or 6/S*A) in cells (n = ∼30 for each). As indicated, we observe that a significantly larger fraction of nuclear 6/S*A (60.0±4.0%, **p<0.005) was localized in the nucleoplasm as compared to that of WT (which is only at 35.5±8.5% of the total). Scale bar represents 10 µm.
(TIF)

Figure S2 Sub-nuclear mobilization of NCL (WT and 6/S*A). U2OS cells were transfected with GFP-NCL (WT and 6/S*A). Post 24 h of transfection, cells were either untreated or treated with CPT and FRAP was performed as described. FRAP analyses suggests GFP-6/S*A mutant is slightly more mobile within the nucleoli with shorter recovery time seen after photobleaching. Although genotoxic stress (treatment with camptothecin, CPT, 2 µM for 2 h) caused greater mobility of both WT and the 6/S*A mutant, the mutant consistently showed higher mobility compared to WT under these conditions. *Statistically different from NCL-WT, p<0.05.
(TIF)

Figure S3 ***In vivo*** **NCL phosphorylation.** ^32^PO_4_ metabolic labeling followed by NCL-IP (immunoprecipitation) resulted in reduced phosphorylation in the presence of CK2 inhibitor DRB. Anti-NCL represents Western blot. Corresponding sub-nuclear localization with GFP-NCL transfection in U2OS cells suggests that NCL mobilization to nucleoplasm is concurrent with hypophosphorylation observed in the presence of CK2 inhibitor.
(TIF)

Figure S4 **NARF6-NCL clones express p14ARF upon IPTG induction and stabilize p53.** NARF6-NCL clones were grown without doxycycline for 15 d and 1 mM IPTG was added for another 22 h. Lysates were obtained from inducible NCL clones (C1 and C2) or Ctrl (vector expressing) clone. Western blots representing 3xFlag-NCL or p14ARF expression upon activation of Tet-off or IPTG-induced promoter, respectively. Both NCL and p14ARF expression increases p53 and its downstream target p21 protein levels. PCNA was used as loading control while NPM (nucleophosmin) was used as nucleolar protein control. ‘+’ indicates with, ‘-’ indicates without. Spliced out and recombined lanes are denoted by a vertical line.
(TIF)

Figure S5 **Half-life analyses of p53 and endogenous NCL upon p14ARF expression.** NARF6 cells were induced for p14ARF expression by 1 mM IPTG treatment for 22 h. The half-life of p53 protein upon robust p14ARF expression is beyond 2 h. Endogenous NCL half-life remains overall unaltered (∼6 h or more) although a transient increase (lane 3 vs. lane 2) upon p14ARF expression is consistently observed. Spliced out and recombined lanes are denoted by a vertical line.
(TIF)

Figure S6 **NARF6-NCL clones as NCL-replacement tool.** Inducible NCL cells grown without doxycycline for 10 d were used to selectively downregulate endogenous NCL. Following two subsequent siRNA transfection and post 36 h of second siRNA treatment, lysates were prepared. Western blot analyses of both endogenous (lower band) and induced NCL (upper band) expression were performed by anti-NCL antibodies to achieve reliable normalized levels. Normalized values against actin using NIH Image J software is represented below each blot. As indicated, up to 70% reduction of endogenous NCL protein was achieved. The expression of induced NCL (i.e. the Flag-tagged NCL) remained unchanged (as indicated by anti-NCL as well as anti-Flag blots).
(TIF)

Figure S7 Half-life analyses of p53 at earlier time points upon inducible NCL (WT or 6/S*A) expression. *Upper panel*, the graph shows evaluation of p53-stability for shorter time period following cycloheximide blocking upon NCL (WT or 6/S*A) expression in the absence of doxycycline (16 d–22 d). As indicated, the p53 half-life is lower in cells expressing WT (∼30–40') as compared to mutant (∼1 h), while control cells have normal half-life of ∼15–20'. *Lower panel*, assuming the decrease in p53 protein levels is a pseudo-first order kinetic process, the data presented in Figure 3B were plotted on a log scale to indicate a higher p53 protein half-life in cells with mutant-NCL expression.
(TIF)

Figure S8 **NCL expressing clones have similar cell properties.** *Upper panel*, the three cell lines (Ctrl, or expressing inducible NCL-WT or NCL-6/S*A) have comparable standard curve as analyzed using MTS assay. *Lower panel*, in the absence of induced NCL expression (i.e. in the presence of doxycycline), all Ctrl, NCL-WT or NCL-6/S*A cells have similar proliferation rates.
(TIF)

Figure S9 Induced NCL-6/S*A expression (10-24 d) activates p53 checkpoint and apoptosis pathway. *Upper panel*, Inducible NCL (WT or 6/S*A) cells were grown without Dx for 10–24 days. Western blots indicating inducible NCL-expression (WT or 6/S*A) by anti-Flag antibodies. Both WT and 6/S*A expression resulted in an increase in p53 and p21 protein levels. However, higher expression of BH3-only pro-apoptotic markers (BID and PUMA) were primarily associated with NCL-6/S*A expression. The quantification was done by NIH Image J software. Values were first corrected for the β-actin levels, then compared to Ctrl cells and indicated below each blot. *Lower panel*, Scatter plots of p53, p21 and BH3-only protein levels shown in upper panel. The data is representative of at least two independent experiments.
(TIF)

Figure S10 **Cumulative expression pattern of markers in the p53 and apoptotic signaling upon NCL expression.** A cumulative scatter plot for all the combined data (with as little as 24 h to as long as 28 days of induced NCL expression) clearly reveals increased levels of p53, BID and PUMA with NCL-6/S*A expression as compared to WT or Ctrl cells These data are derived from multiple Westerns and presented as combined plots for the p53, p21 and BH3-only pro-apoptotic markers (BID, BIM and PUMA) protein levels. The quantification was done by NIH Image J software. Values were first corrected for the β-actin levels and then compared to Ctrl (no exogenous NCL, no Dx day 1 or 7) cells. The graph is representative of at least two independent experiments.
(TIF)
