# Supplementary figures and images for: Induced Expression of Nucleolin Phosphorylation-Deficient Mutant Confers Dominant-Negative Effect on Cell Proliferation
Source: PLoS One. 2014 Oct 14;9(10):e109858. doi: 10.1371/journal.pone.0109858 (PMC4196967; doi:10.1371/journal.pone.0109858)

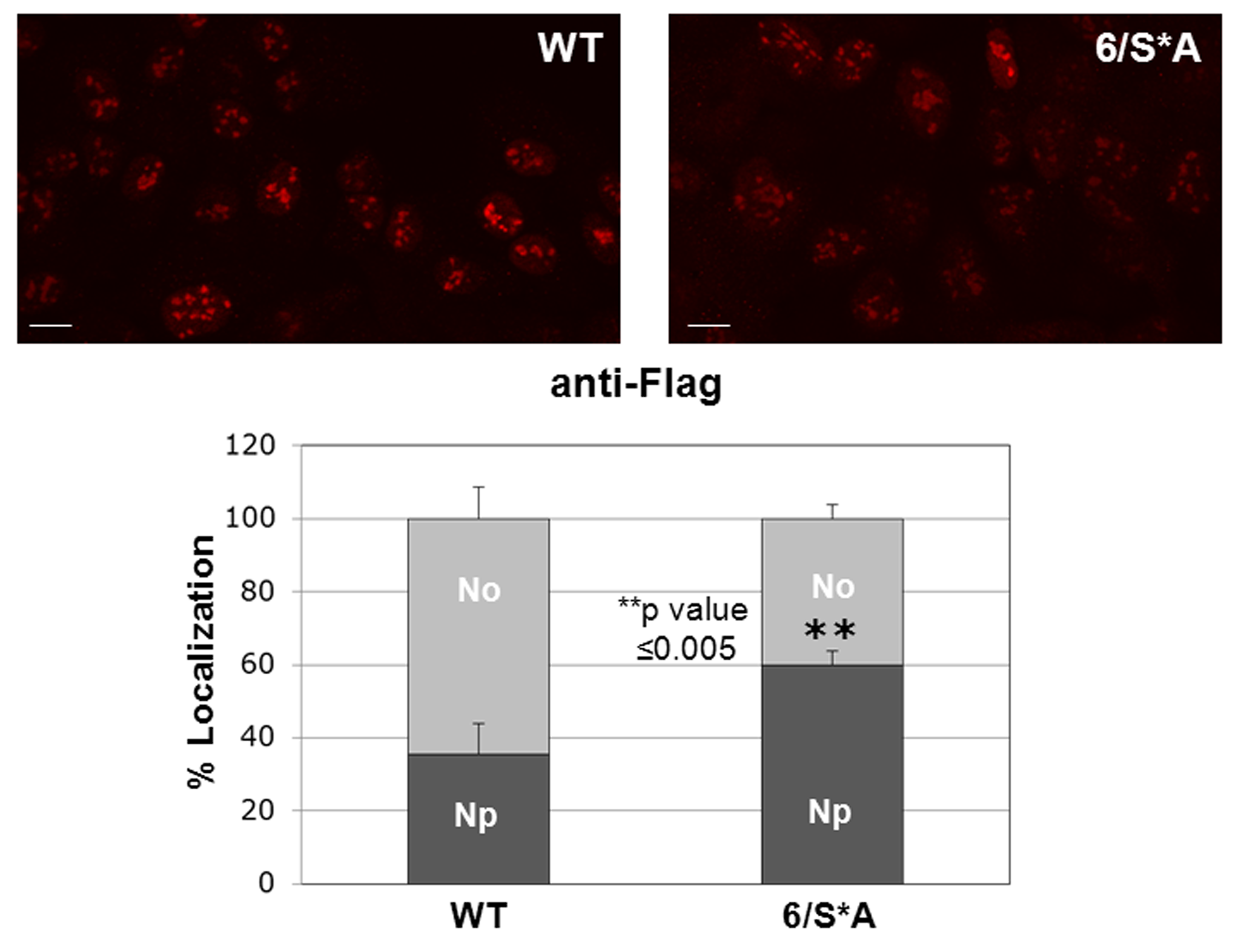

Supplement: Figure S1 — Sub-nuclear distribution of NCL (WT and 6/S*A). Inducible NCL cells grown without doxycycline for 15–29d were used to detect NCL by immunofluorescence using anti-Flag antibodies. Image acquisition was done with constant parameters between the samples. Integrated Morphometry Analysis (IMA) was performed for ∼80–100 nuclei as described. Upper panel is representative image at moderate level of NCL expression. The graph represents sub-nuclear distribution of moderately expressed NCL (WT or 6/S*A) in cells (n = ∼30 for each). As indicated, we observe that a significantly larger fraction of nuclear 6/S*A (60.0±4.0%, **p<0.005) was localized in the nucleoplasm as compared to that of WT (which is only at 35.5±8.5% of the total). Scale bar represents 10 µm. (TIF) [file pone.0109858.s001.tif]

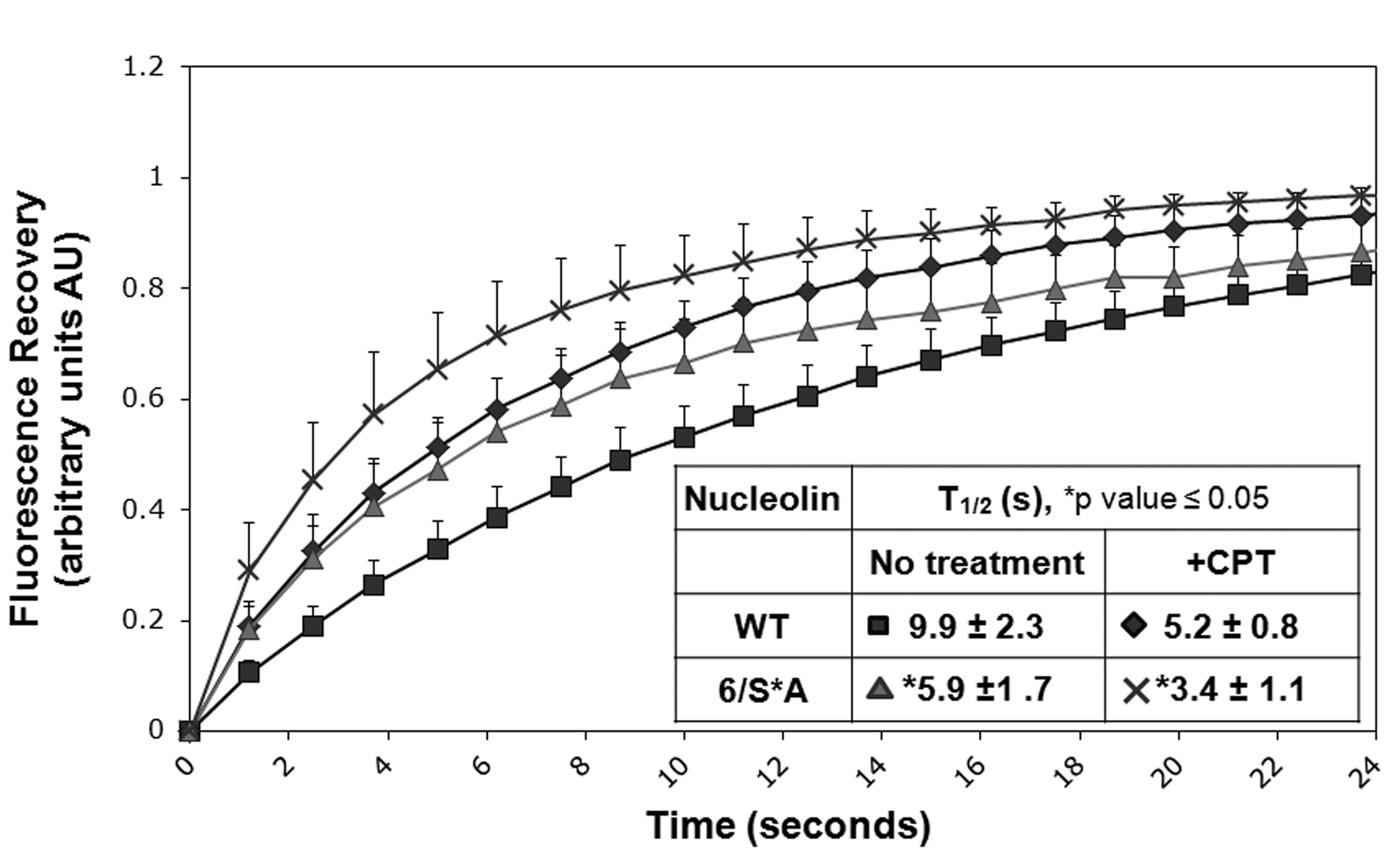

Supplement: Figure S2 — Sub-nuclear mobilization of NCL (WT and 6/S*A). U2OS cells were transfected with GFP-NCL (WT and 6/S*A). Post 24 h of transfection, cells were either untreated or treated with CPT and FRAP was performed as described. FRAP analyses suggests GFP-6/S*A mutant is slightly more mobile within the nucleoli with shorter recovery time seen after photobleaching. Although genotoxic stress (treatment with camptothecin, CPT, 2 µM for 2 h) caused greater mobility of both WT and the 6/S*A mutant, the mutant consistently showed higher mobility compared to WT under these conditions. *Statistically different from NCL-WT, p<0.05. (TIF) [file pone.0109858.s002.tif]

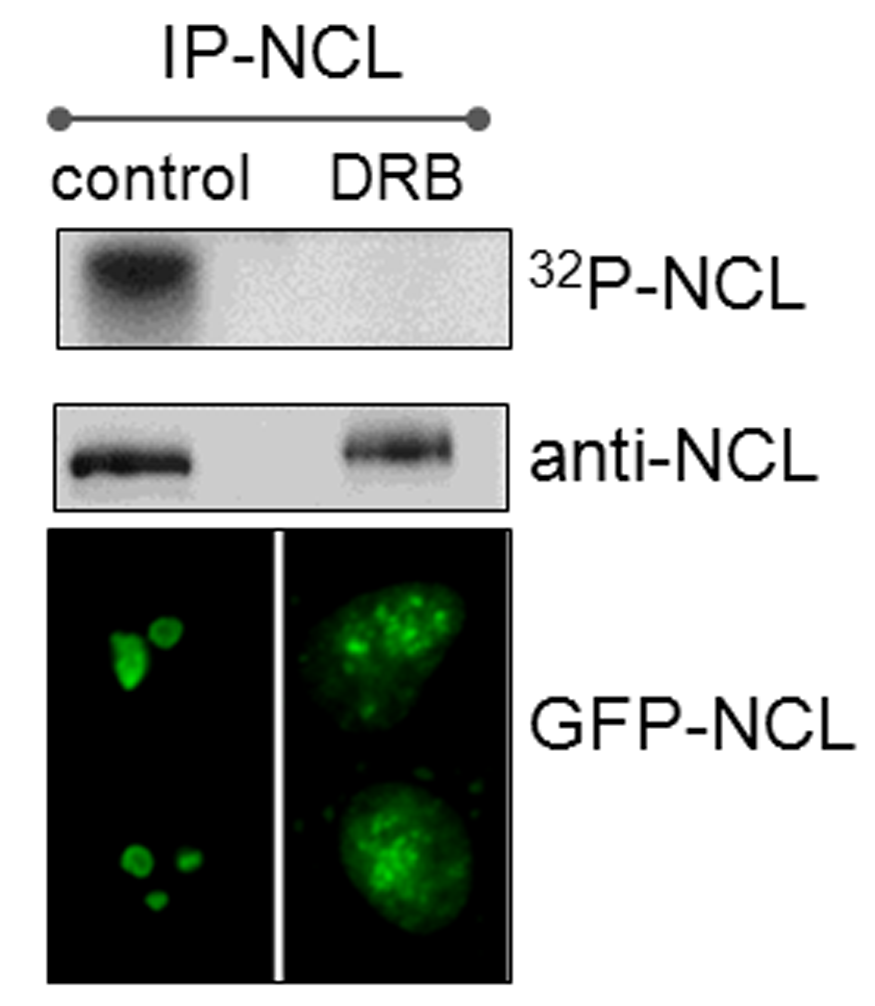

Supplement: Figure S3 — In vivo NCL phosphorylation. 32PO4 metabolic labeling followed by NCL-IP (immunoprecipitation) resulted in reduced phosphorylation in the presence of CK2 inhibitor DRB. Anti-NCL represents Western blot. Corresponding sub-nuclear localization with GFP-NCL transfection in U2OS cells suggests that NCL mobilization to nucleoplasm is concurrent with hypophosphorylation observed in the presence of CK2 inhibitor. (TIF) [file pone.0109858.s003.tif]

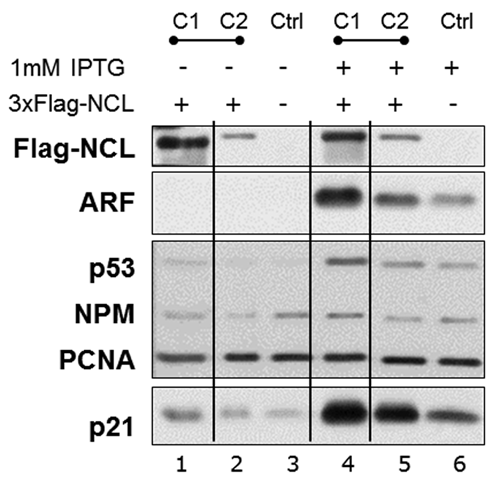

Supplement: Figure S4 — NARF6-NCL clones express p14ARF upon IPTG induction and stabilize p53. NARF6-NCL clones were grown without doxycycline for 15 d and 1 mM IPTG was added for another 22 h. Lysates were obtained from inducible NCL clones (C1 and C2) or Ctrl (vector expressing) clone. Western blots representing 3xFlag-NCL or p14ARF expression upon activation of Tet-off or IPTG-induced promoter, respectively. Both NCL and p14ARF expression increases p53 and its downstream target p21 protein levels. PCNA was used as loading control while NPM (nucleophosmin) was used as nucleolar protein control. ‘+’ indicates with, ‘-’ indicates without. Spliced out and recombined lanes are denoted by a vertical line. (TIF) [file pone.0109858.s004.tif]

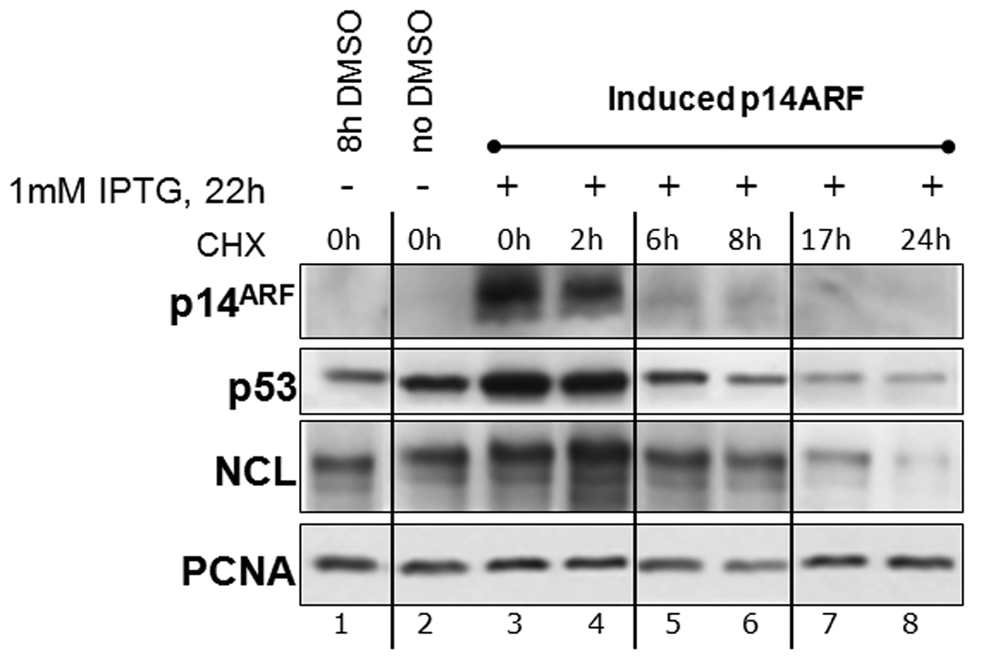

Supplement: Figure S5 — Half-life analyses of p53 and endogenous NCL upon p14ARF expression. NARF6 cells were induced for p14ARF expression by 1 mM IPTG treatment for 22 h. The half-life of p53 protein upon robust p14ARF expression is beyond 2 h. Endogenous NCL half-life remains overall unaltered (∼6 h or more) although a transient increase (lane 3 vs. lane 2) upon p14ARF expression is consistently observed. Spliced out and recombined lanes are denoted by a vertical line. (TIF) [file pone.0109858.s005.tif]

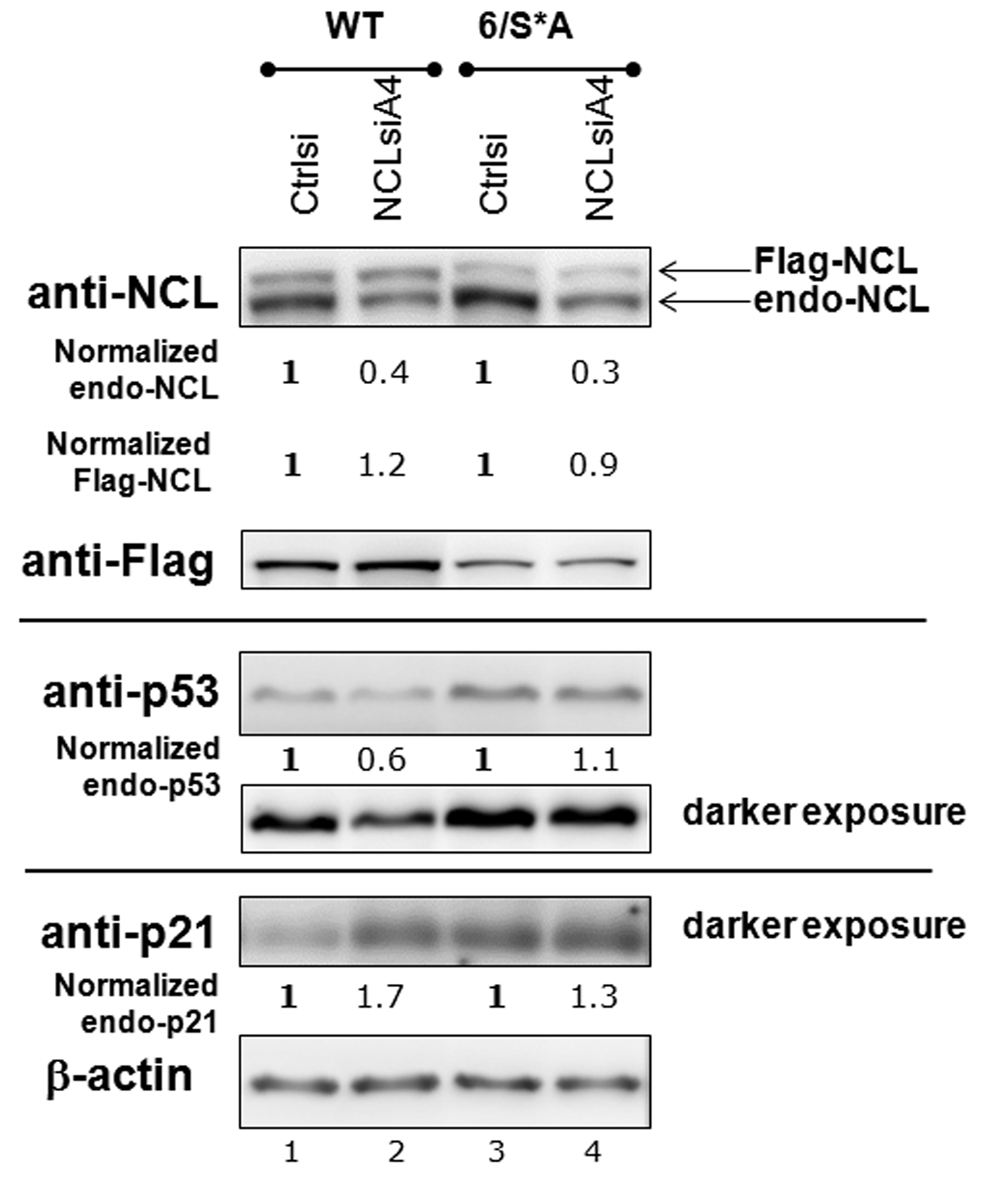

Supplement: Figure S6 — NARF6-NCL clones as NCL-replacement tool. Inducible NCL cells grown without doxycycline for 10 d were used to selectively downregulate endogenous NCL. Following two subsequent siRNA transfection and post 36 h of second siRNA treatment, lysates were prepared. Western blot analyses of both endogenous (lower band) and induced NCL (upper band) expression were performed by anti-NCL antibodies to achieve reliable normalized levels. Normalized values against actin using NIH Image J software is represented below each blot. As indicated, up to 70% reduction of endogenous NCL protein was achieved. The expression of induced NCL (i.e. the Flag-tagged NCL) remained unchanged (as indicated by anti-NCL as well as anti-Flag blots). (TIF) [file pone.0109858.s006.tif]

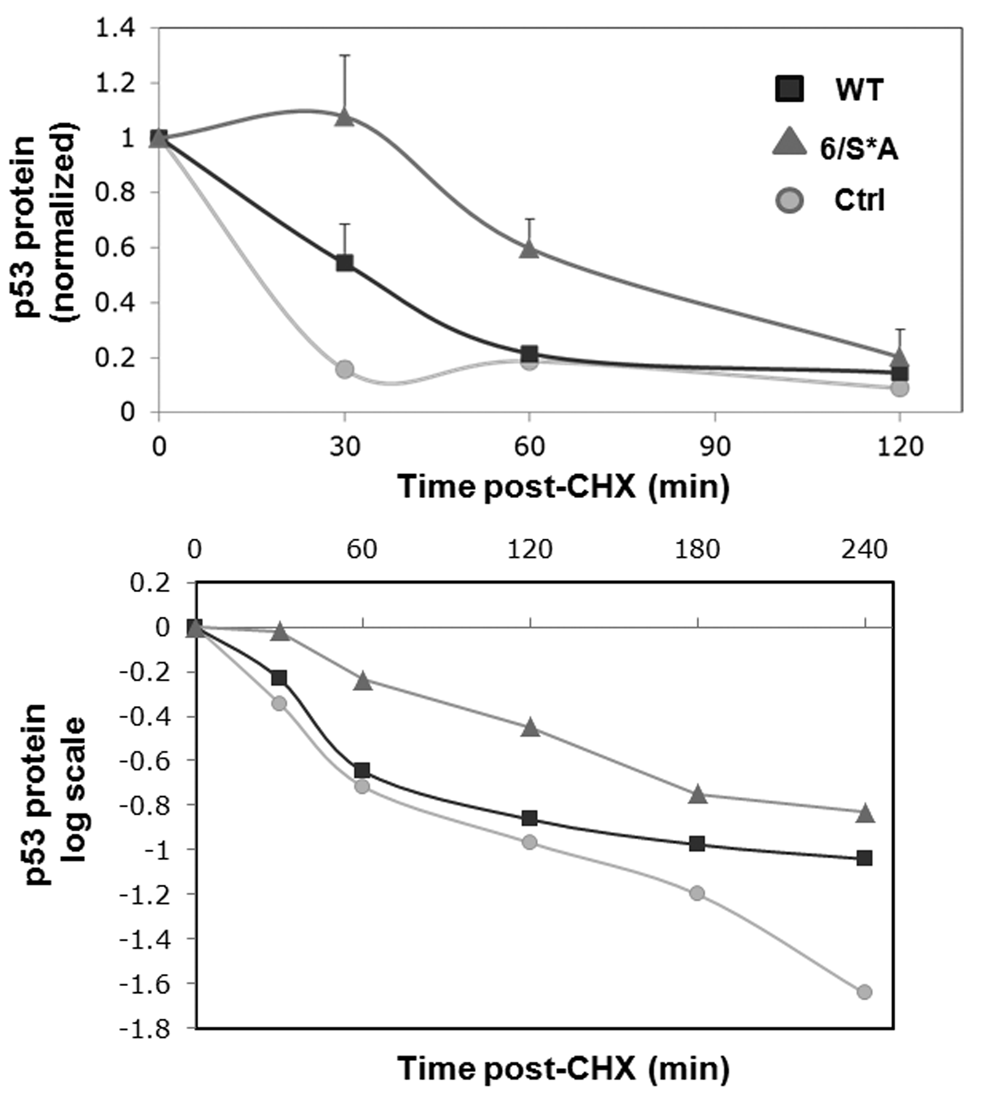

Supplement: Figure S7 — Half-life analyses of p53 at earlier time points upon inducible NCL (WT or 6/S*A) expression. Upper panel, the graph shows evaluation of p53-stability for shorter time period following cycloheximide blocking upon NCL (WT or 6/S*A) expression in the absence of doxycycline (16 d–22 d). As indicated, the p53 half-life is lower in cells expressing WT (∼30–40') as compared to mutant (∼1 h), while control cells have normal half-life of ∼15–20'. Lower panel, assuming the decrease in p53 protein levels is a pseudo-first order kinetic process, the data presented in Figure 3B were plotted on a log scale to indicate a higher p53 protein half-life in cells with mutant-NCL expression. (TIF) [file pone.0109858.s007.tif]

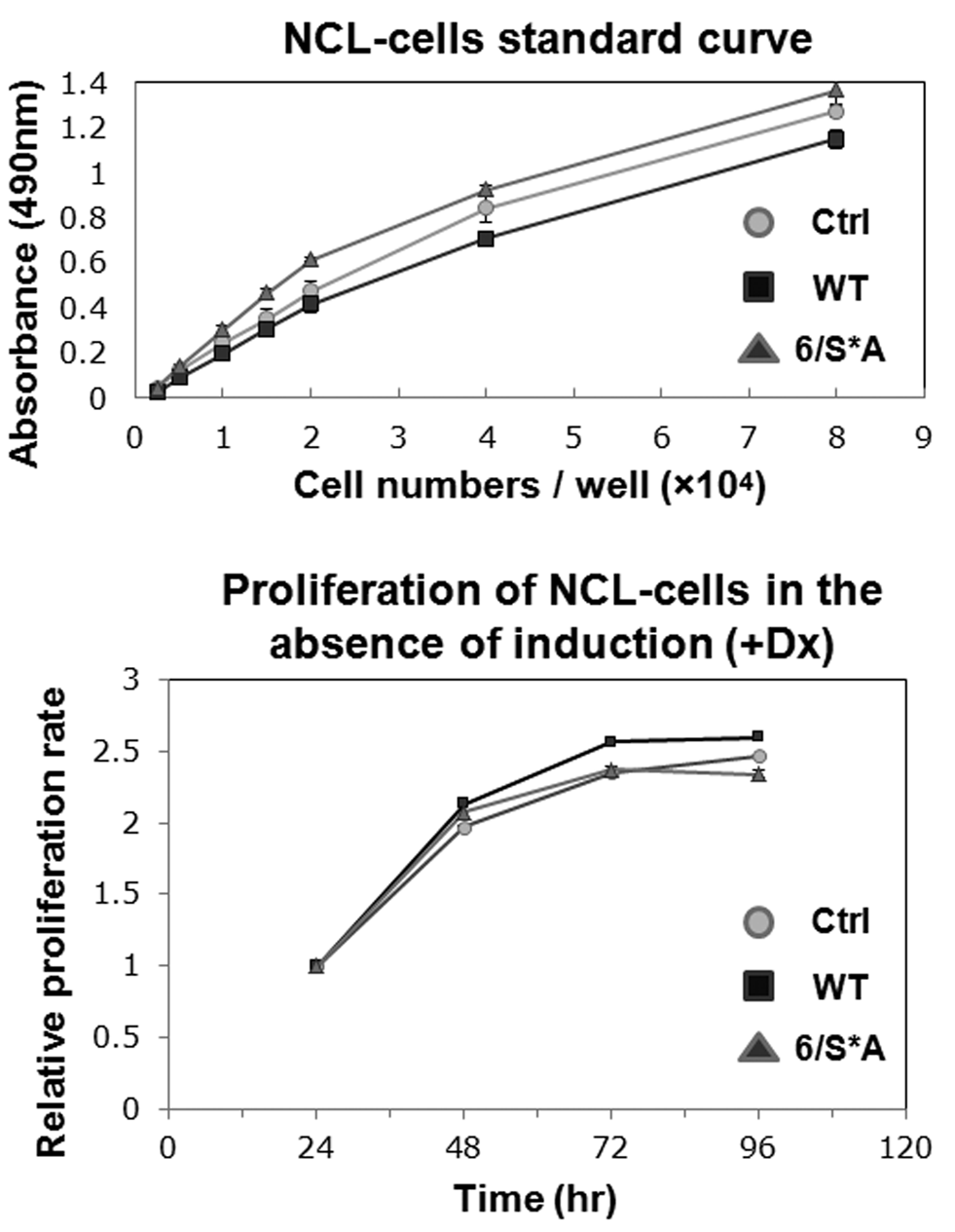

Supplement: Figure S8 — NCL expressing clones have similar cell properties. Upper panel, the three cell lines (Ctrl, or expressing inducible NCL-WT or NCL-6/S*A) have comparable standard curve as analyzed using MTS assay. Lower panel, in the absence of induced NCL expression (i.e. in the presence of doxycycline), all Ctrl, NCL-WT or NCL-6/S*A cells have similar proliferation rates. (TIF) [file pone.0109858.s008.tif]

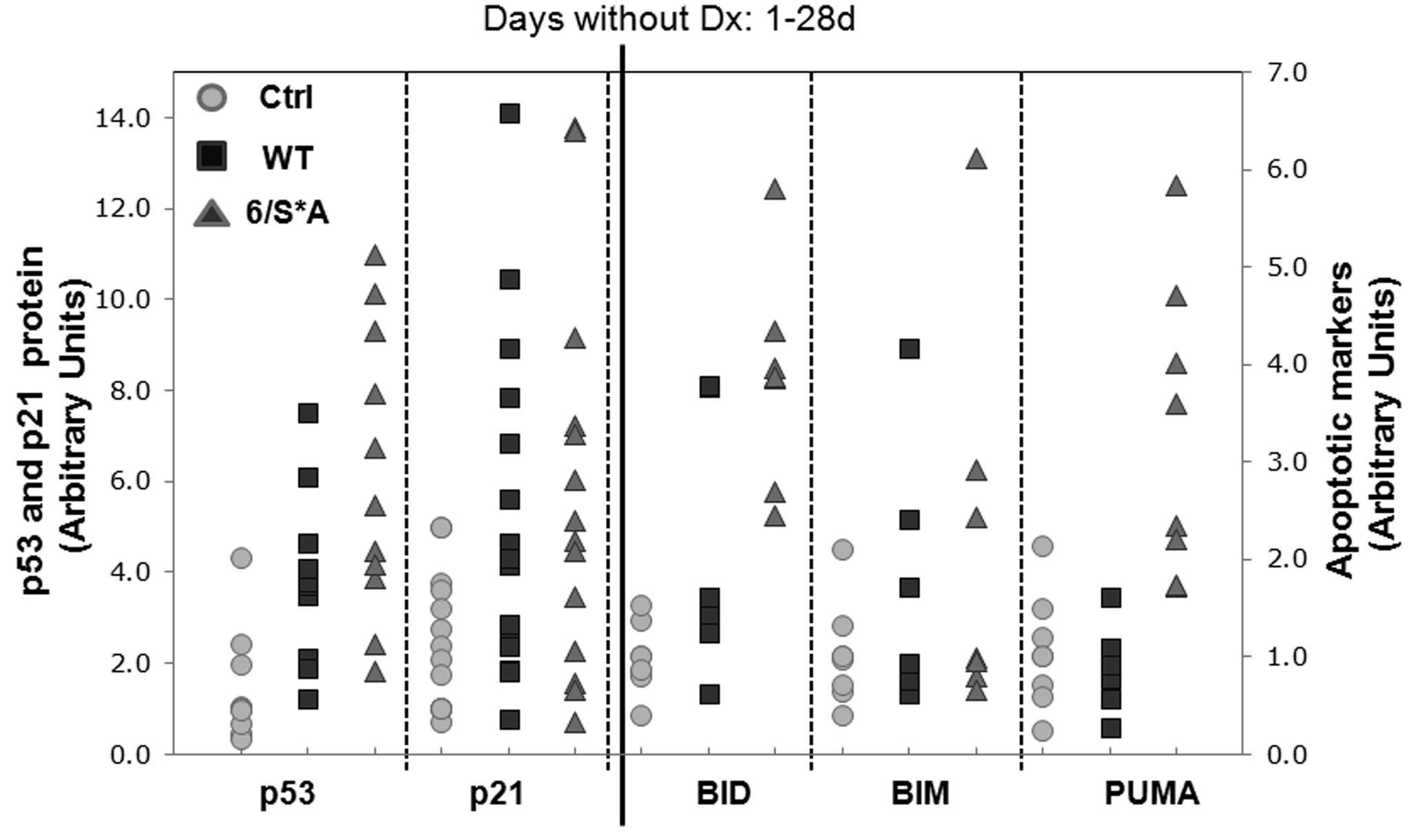

Supplement: Figure S10 — Cumulative expression pattern of markers in the p53 and apoptotic signaling upon NCL expression. A cumulative scatter plot for all the combined data (with as little as 24 h to as long as 28 days of induced NCL expression) clearly reveals increased levels of p53, BID and PUMA with NCL-6/S*A expression as compared to WT or Ctrl cells These data are derived from multiple Westerns and presented as combined plots for the p53, p21 and BH3-only pro-apoptotic markers (BID, BIM and PUMA) protein levels. The quantification was done by NIH Image J software. Values were first corrected for the β-actin levels and then compared to Ctrl (no exogenous NCL, no Dx day 1 or 7) cells. The graph is representative of at least two independent experiments. (TIF) [file pone.0109858.s010.tif]
